# Enterovesical Fistula Secondary to Transitional Cell Carcinoma of the Bladder

**DOI:** 10.1055/s-0038-1673663

**Published:** 2018-10-18

**Authors:** Mark Bugeja, Christine Mizzi, Ernest Ellul, Simon Bugeja, Stephen Mattocks

**Affiliations:** 1 Department of Surgery, Mater Dei Hospital, Msida, Malta; 2 Department of Urology, Mater Dei Hospital, Msida, Malta

**Keywords:** Enterovesical fistual, bladder cancer, transitional cell carcinoma

## Abstract

Although fistulae between the urinary bladder and the gastrointestinal tract are not uncommon, those caused by carcinoma of the urinary bladder are rare. This report describes the case of an 85 years old male who was diagnosed with a mass involving the small bowel and the urinary bladder during the course of investigation for recurrent urinary tract infections. At laparotomy, the presence of an enterovesical fistula involving the ileum and bladder was confirmed. Histopathological examination of the resected mass showed poorly differentiated urothelial carcinoma. No early postoperative complications were encountered and postoperative cystography showed healing of the bladder without evidence of leakage. Due to the patient's age and comorbidities, no further oncological treatment was offered. Three months later the patient was readmitted to hospital with a severe pneumonia to which he succumbed.

Enterovesical fistula is a pathological passage between part of the intestine and the bladder. 1 2 3 The first ever enterovesical fistula described in the literature dates back to 200 AD; an individual passing urine per rectum. 4 It was in the late 19 ^th^ century that enterovesical fistulae became more frequently reported. 4 Enterovesical fistulae account for 1 out of every 3,000 surgical admissions. 4 Fistulae between the gastrointestinal tract and the urinary bladder are most often involve the colon. Enterovesical fistulae mostly occur secondary to inflammatory bowel disease. 1 2 3 5 6 However, enterovesical fistulae secondary to primary bladder cancer are extremely rare. 1 2 3 5 6 The diagnosis of an enterovesical fistula can pose a great challenge to the clinician, with the consequence of it being diagnosed several months after symptoms initiate. 1 Here we report a case of an enterovesical fistula secondary to transitional cell carcinoma (TCC) of the bladder fistulating into the ileum and presenting with recurrent urinary tract infections.

## Case Report

An 85-year-old gentleman, known to suffer from hypertension and congestive heart failure but with no previous history of abdominal surgery, was admitted to hospital with a 3-week history of dysuria, severe urinary frequency, nocturia, suprapubic pain, fever, and increasing lethargy. He had been treated with antibiotics by his family doctor but symptoms failed to resolve. Urinalysis was indicative of a urinary tract infection with positive nitrites, proteinuria and leukocyturia but urine culture was negative. Blood tests revealed neutrophilia and mild acute renal impairment. The patient was admitted to a medical ward, started empirically on intravenous Ciprofloxacin, and discharged 2 days later on oral antibiotics, and a urological review was arranged.

**Fig. 1:**
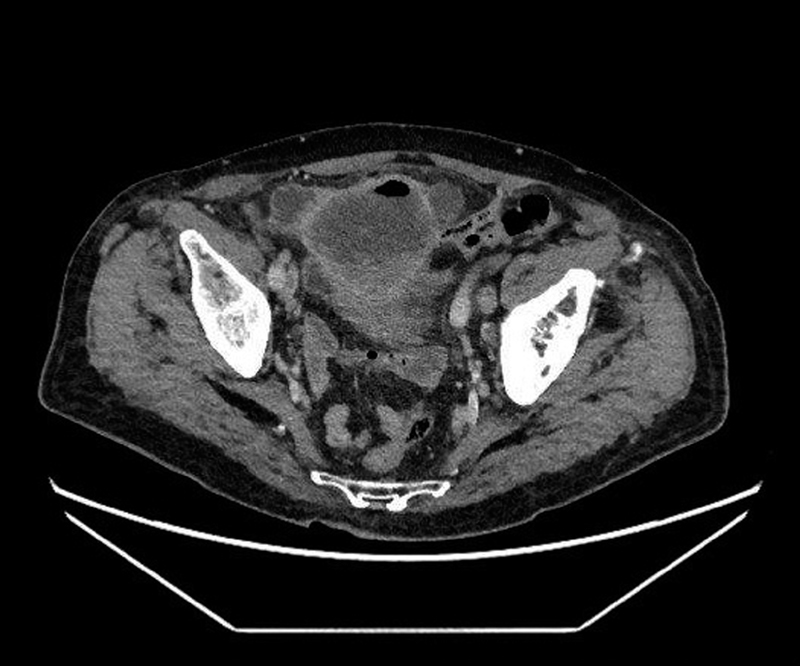
Computed Tomography image of complex mass involving the urinary bladder and small intestine.

He was readmitted 10 days later through emergency department Accident and Emergency (A&E) department, his symptoms persisting. At this point, he was referred to our unit. Again, no bacteria were cultivated from urine. Intravenous Co-Amoxiclav therapy was initiated. In view of these recurrent symptoms, an ultrasound examination of the urinary tract was performed. This showed a heterogenous mass containing gas, anterior to the urinary bladder measuring 8.5 × 4.9 × 6.1cm. To further characterize the findings on ultrasound examination, a computed tomography (CT) of the thorax, abdomen, and pelvis with intravenous contrast was requested. This showed a large, thick walled mass measuring 8cm superior to the urinary bladder, containing fluid and gas, communicating with the urinary bladder, and in contact with small bowel ( Fig. 1 ). Several enlarged lymph node groups, namely in both groins, lateral vesical groups bilaterally and left para-aortic region up to the level of the left renal hilum were noted.

A urinary catheter was inserted and ‘cloudy’ urine containing a sediment drained. Cystoscopy was performed primarily to localize the fistula and its relation to the ureteric orifices in preparation for extirpative surgery. At cystoscopy, the bladder was found to be full of debris and a fistulous opening was seen well away from both ureteric orifices. A Gastrografin enema of the rectum showed no leakage of contrast material from the colon into the urinary bladder. *Klebsiella oxytoca* and *Enterococcus faecium* were eventually cultivated from further microbiological examination of the urine.

**Fig. 2:**
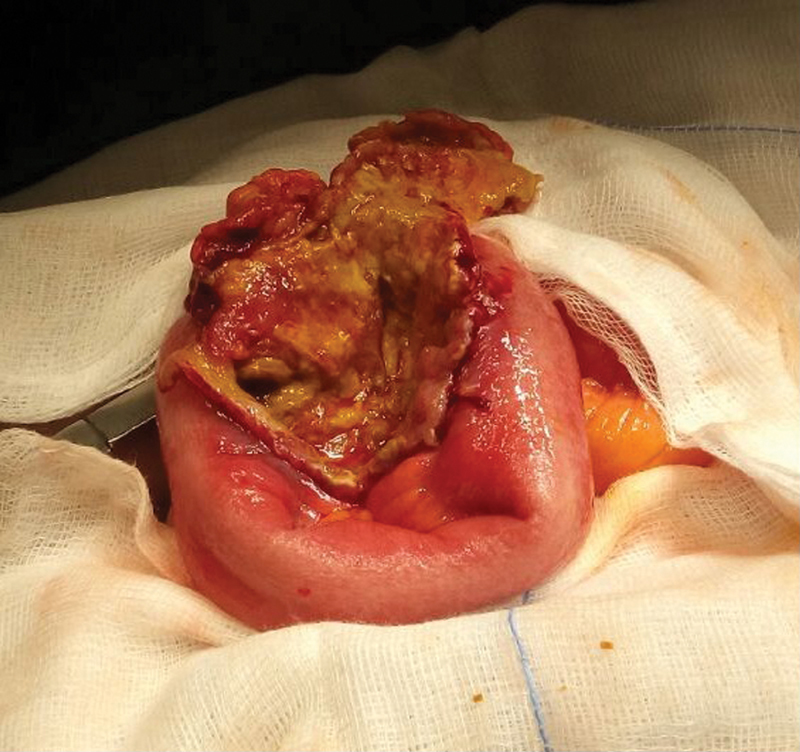
Specimen showing small intestine and dome of urinary bladder, opened after excision of specimen.

The patient underwent laparotomy, during which a complex mass involving the proximal ileum and fundus of urinary bladder together with enlarged lymph nodes in the left obturator-inguinal region were found. The small bowel mass involving a wide fistula (approximately 3 cm diameter) was dissected off the dome of the bladder ( Fig. 2 ). The involved loop of ileum was resected and a stapled (GIA80 Covidien). Primary anastomosis was performed. A partial cystectomy was possible, circumscribing the fistula while leaving an adequate capacity bladder and preserving the trigone. A suprapubic urinary catheter and a urethral catheter were inserted for bladder drainage in the postoperative period which proved to be uneventful. A cystogram performed 3 weeks after the operation showed no leakage of urine and the catheters were removed. The patient was discharged from hospital a few days later, the total hospital stay being 5 weeks. Minimal discomfort was reported from urinary symptoms on discharge.

Histopathological examination of the resected tissues was reported as showing poorly differentiated urothelial carcinoma (pT4b). Both vascular and perineural invasion were identified. The tumor was transmural in the bladder specimen and ulcerated through the mucosa of the ileum. Resection margins were reported to be clear of tumor. Immunohistochemistry showed strong CK7, MNF116, and p63 expression. There was focal S-100 expression. There was no expression of CK20, CDX2, or Desmin.

Further to an oncologist evaluation, no further treatment was planned due to the patient's advanced age and comorbidities. He was hospitalized again 3 months after discharge suffering from a severe pneumonia to which he succumbed.

## Discussion

Fistulation between the gastrointestinal tract and the lower urinary tract is not uncommon. Most frequently, these originate from a pathological process in the gastrointestinal tract with a urological cause being much less frequent. Fistulae originating from the urinary bladder are said to account for only 4.6% of cases. 7 Inflammation (52%) is the most common pathological process giving rise to an enterovesical fistula followed by neoplasia (35%) and trauma (16%). 2 3 8

Fistulae arising from complications of diverticular disease account for 65 to 79% of cases. 1 9 They are almost exclusively colovesical. Enterovesical fistulae secondary to Crohn's disease account for 5 to 7% of cases with an ileovesical fistula being the commonest type. 1 9 Similarly, Meckel's diverticulum, ileal tumors and appendicular abscess have all been reported as causes of enterovesical fistulae. 10 11 12 Iatrogenic fistulae occur as a consequence of general surgical, vascular, and urological interventions, or as a complication of chemotherapy and radiotherapy. 1 9 Those secondary to trauma include urethral catheterisation penetrating abdominal and pelvic injuries and foreign bodies in the bowel, urinary bladder, or peritoneal cavity. 13 14 15

Advanced colon and bladder malignancies account for up to one-fifth of all cases, with the latter being extremely rare. 1 Only 5% of all enterovesical fistulae are due to bladder carcinoma. 16 Other rare urological causes include a bladder diverticulum erosion of a large bladder calculus or as a complication of a prostate abscess. 7

Over 75% of patients with an enterovesical fistula present with pneumaturia, faecaluria, and recurrent urinary tract infections. Faecaluria is pathognomonic. 3 7 This is due to high bladder wall compliance which contributes to low intravesical pressures, thus favoring flow from bowel to bladder. 4 Passage of urine per rectum is reported in just approximately 16% of cases. 3 7 This is in contrast with urorectal fistulae where patients present with passage of urine per rectum. Thus, in enterovesical fistulae bowel diversion rarely controls symptoms unless pelvic sepsis is present. 17

The diagnosis of enterovesical fistula is predominantly a clinical one. Functional studies, such as the charcoal test where charcoal is detected in the urine after ingestion of nonabsorbable charcoal can be performed and are positive in between 80 to 100% of cases. 3 However, they do not provide localizing anatomic information. Computed Tomography (CT) is the radiological investigation of choice in establishing the site, complexity of the fistula and identification of the etiological factor. Oral administration of contrast permits detection of contrast in the bladder. Together with the detection of free air in the bladder, this is a pathognomonic finding. 1 Magnetic Resonance Imaging (MRI), although not used routinely, has been advised by some authors because of its excellent intrinsic soft tissue resolution. It has a multiplanarimaging capability allowing for an accurate definition of the fistulous tract, something which is not always possible with CT scan. 1 18

Cystoscopy can be helpful in diagnosis. 5 Biopsy can be taken to diagnose malignancy. Indirect evidence, such as localized erythema and papillary/bullous changes in the mucosa is found in approximately 90% of cases. 19 Occasionally, material oozing through an opening is present. The omission of a biopsy from the edge of the fistula during cystoscopy, in this case, meant that etiology was not confirmed prior to surgery. Nevertheless surgery had been planned as definitive treatment, if only, to alleviate his symptoms. Therefore, preoperative histological diagnosis would have not altered the management strategy. Additionally, in many cases with extensive inflammatory change and tissue necrosis, despite cystoscopy and biopsy, the cause of enterovesical fistula remains elusive and is only established at laparotomy. 2

When a bladder tumor presents as an enterovesical fistula, it is by definition a stage 4 tumor. Therefore, complete surgical excision of the bladder malignancy with en bloc resection of the involved segment of small bowel is the only surgical procedure which can be performed with a curative intent. Yet, prognosis remains relatively poor with an overall 44% 5 years and a 23% 10 years of survival. 20 However, the symptomatology is often so devastating that palliative surgical treatment is still attempted in most cases. 2

The scope of this case report is not to report the enterovesical fistula per se. As described above, enterovesical fistulae are not infrequently occurred. Our aim is to specifically highlight the cause of the pathology–a very rare cause. Indeed to our knowledge, only two cases of enterovesical fistulae secondary to transitional cell bladder carcinoma have been published in Western literature. These are the cases reported by Reig Ruiz et al (1994) 7 and Dawam et al. 6 While the case reported by Dawam et al 6 was very similar to this case, the patient reported by Reig Ruiz et al 7 presented with different symptoms, namely, profuse diarrhea and anuria. Moreover, there was a previous history of surgery for intestinal perforation from typhoid. A significant clinical finding was the presence of an extraluminal mass on digital rectal examination. Another two cases of enterovesical fistulae secondary to urinary bladder carcinoma have been reported by Sellers and Fiorelli 2 and Yang et al 5 but these were secondary to squamous cell carcinoma of the urinary bladder.

## Conclusion

Enterovesical fistulae secondary to transitional cell carcinoma of the urinary bladder are extremely rare, with only two cases published in Western literature. Not only are they rare but they are also difficult to diagnose. Thus, we recommend that a diagnosis of enterovesical fistula should always be considered in patients presenting with suggestive symptoms.
